# A possible injectable tissue engineered nucleus pulposus constructed with platelet-rich plasma and ADSCs in vitro

**DOI:** 10.1186/s13018-020-01840-1

**Published:** 2020-08-08

**Authors:** ZhiCheng Zhang, Jian Ma, DaJiang Ren, Fang Li

**Affiliations:** 1 Department of Orthopedic, the Seventh Medical Center of Chinese PLA General Hospital, Beijing, 100700 People’s Republic of China; 2 Department of Orthopedic, the First People’s Hospital of Yangquan City, Yangquan City, Shanxi Province 045000 People’s Republic of China

**Keywords:** Platelet-rich plasma gel, Adipose-derived stem cells, Tissue engineering, Nucleus pulposus

## Abstract

**Background:**

Injectable tissue engineered nucleus pulposus is a new idea for minimally invasive repair of degenerative intervertebral disc. The platelet-rich plasma (PRP) and adipose-derived stromal cells (ADSCs) could be harvested from autologous tissue easily. PRP contains numerous autologous growth factors and has reticulate fibrous structure which may have the potential to make ADSCs differentiate into nucleus pulposus-like cells. The goal of this study was to explore the feasibility of constructing a possible injectable tissue engineered nucleus pulposus with PRP gel scaffold and ADSCs.

**Methods:**

After identification with flow cytometry, the rabbit ADSCs were seeded into PRP gel and cultured in vitro. At the 2nd, 4th, and 8th week, the PRP gel/ADSCs complex was observed by macroscopy, histological staining, BrdU immunofluorescence, and scanning electron microscopy. The glycosaminoglycans (GAG) in the PRP gel/ADSCs complex were measured by safranin O staining with spectrophotometry. In PRP gel/ADSCs complex, gene expression of HIF-1α, aggrecan, type II collagen were tested by RT-PCR. The injectability of this complex was evaluated.

**Results:**

Macroscopically, the complex was solidified into gel with smooth surface and good elasticity. The safranin O dye was almost no positive staining at 2nd week; however, the positive staining of extracellular matrix was enhanced obviously at 4th and 8th week. The HE staining and SEM demonstrated that the cells were well-distributed in the reticulate scaffold. BrdU immunofluorescence showed that ADSCs can survive and proliferate in PRP gel at each time points. The level of GAG at 4th week was higher than those at 2nd week (*P* < 0.05), and significant difference was also noted between 4th and 8th week (*P* < 0.05). HIF-1α, aggrecan, type II collagen gene expression at 4th week were much more than those at 2nd week (*P* < 0.05), and significant differences were also noted between 4th and 8th week (*P* < 0.05). The flow rate of complex was 0.287 mL/min when passed through the 19-gauge needle with the 100 mmHg injection pressure.

**Conclusions:**

Our preliminary findings suggest that the PRP gel make it possible for rabbit ADSCs differentiated into nucleus pulposus-like cells after coculture in vitro. According to the results, it is a better feasible method for construction of autologous injectable tissue engineered nucleus pulposus.

## Background

In recent years, the minimally invasive techniques have been widely applied in spinal surgery. It has made the percutaneous application of injectable tissue engineered nucleus pulposus possible to repair the degenerative intervertebral disc. Accordingly, the researches of injectable tissue engineered nucleus pulposus become increasingly notable and showing widely prospect of clinical application. Many nature-derived materials such as collagen gel, fibrin glue, and chitosan were also developed as injectable carriers for mesenchymal stem cells (MSCs), which shown better biocompatibility and biodegradability. Platelet-rich plasma (PRP) is blood plasma that has been enriched with platelets which is obtained by sequestering and concentrating platelets by gradient density centrifugation [1]. As a concentrated source of autologous platelets, PRP releases several different growth factors and other cytokines through degranulation that promote healing of bone and soft tissue. PRP biological effects depend on the activation of α-granules in platelet resulting in release of various high concentrative growth factors and depends on the reticulate fibrous cross-linking structure from fibrinogen [2]. Formed reticulate fibrous scaffold can support new tissues regeneration with various growth factors induction. The release of growth factors from PRP is extremely trifling before activated. The coagulant such as calcium and thrombin may play the important role as activator and induce immediate growth factors release in a dose-dependent fashion [3].

PRP has been an emerging biologic tool in orthopedic surgery and regenerative medicine [4, 5]. Initially, PRP was studied in bone repair as a method of improving union rate based on the pioneering work of Marx et al. [6]. In this study, PRP was mixed with bone grafts to repair the bone defects in oral and maxillofacial surgery. The group of bone grafts with PRP acquired higher radiographic union rate and bone density than that of grafts without PRP. They found that the mechanism of this osteogenesis promotion may be the high concentration of platelet-derived growth factor (PDGF) and transforming growth factor-β1 (TGF-β1). Furthermore, PRP may be contributive to initiation, enhancement, or acceleration of soft tissue healing. As a phenomenon, the first physical response to soft tissue injury is to deliver platelets. In the process of soft tissue repair, platelets may recruit the critical assistance of stem cells. PRP is currently being used to treat acute and chronic tendinopathies in the clinical and surgical settings [7, 8]. It also had been certificated by several studies that PRP could improve wound healing in total knee replacements and rotator cuff injuries [9–11]. Recently, the clinical injection application of PRP is widespread in various chronic tendinopathies [8, 12] which make injectable PRP gel scaffold possible.

In fact, the most significant advantage of PRP over other potential therapies is autologous. PRP contains numerous autologous growth factors such as TGF-β1, PDGF, and insulin-like growth factor (IGF) [13], which have been proven to improve the proliferation of MSCs. Recent research also observed a proliferative effect for MSCs exposed to PRP in monolayer culture and an increase in the expression of chondrogenic markers when cells were cocultured in the 3D environment [14]. This make it possible that autologous ADSCs differentiate into nucleus pulposus-like cells after coculture in vitro [15]. Moreover, PRP gel is a wonderful scaffold to induce osteogenesis. The PRP gel mainly contains fibrillar material with striated band similar to fibrin filaments and platelets. The addition of MSCs in PRP gel scaffold significantly increased new bone formation, mineralization, and mechanical property compared to the PRP-solo group [16]. Then, PRP gel may be an ideal injectable scaffold material for construction of tissue engineered nucleus pulposus. The aim of this research was to explore the feasibility of PRP gel scaffold and ADSCs complex to build injectable tissue engineered nucleus pulposus in vitro. Good results in this research may indicate that PRP gel scaffold and ADSCs could serve as a highly effective and less invasive injectable autologous nucleus pulposus substitute, which could be utilized in many patients with disc degeneration diseases in clinical practice.

## Materials and methods

### Isolation, culture, and BrdU label of ADSCs

The adipose tissue was obtained from the interscapular region of New Zealand rabbits. These tissues were dealt with for ADSCs isolation and culture as previous study described [17]. Briefly, the envelope, connective tissue and small blood vessels of adipose tissue were removed. These adipose tissues were rinsed with phosphate-buffered saline (PBS) containing 1% penicillin and streptomycin, minced into small pieces, and then incubated in a solution containing 0.075% collagenase type IA (Sigma) for 1 h at 37 °C with vigorous shaking. The top lipid layer was removed and the residual liquid was centrifuged with 1500 r/min for 10 min at room temperature. The cell pellets were dealt with erythrocyte lysis buffer for 10 min to lyse red blood cells. The residuary cells were suspended in Dulbecco’s modified Eagle’s medium (DMEM) with 10% fetal bovine serum (FBS, Gibco), and were filtered through a 40-μm cell strainer, and plated in 10-cm petri dish. After reaching 90% confluence, the third generation of ADSCs was harvested and cryopreserved in liquid nitrogen. For cell tracking, ADSCs were labeled with BrdU for 48 h before seeded in PRP.

### Identification of ADSCs

Fluorescence-activated cell sorting (FACS) was used to analyze the surface markers of ADSCs. The cells were placed into Eppendorf tube with 1 × 10^6^cells per 1.0 mL, washed twice with PBS and incubated for one hour at room temperature with the following FITC-conjugated antibodies (Abcam): anti-rabbit D90(1:200), Anti-rabbit CD45(1:200). The samples were then washed twice with PBS and analyzed by FASC.

### Preparation of PRP

The peripheral blood (5 mL) was drawn from rabbit’s central ear artery, then put it into 5 mL EDTA anticoagulation tube. After above procedures, the blood was subjected to centrifugation for 10 min at 2400 rpm, and the obtained supernatant was transferred to another tube. After that, the supernatant was centrifuged for 15 min at 3600 rpm to obtain platelet-poor plasma (PPP) and PRP. The top layer, which consisted of the PPP, was aspirated out and put into a new tube. Then, about 0.5 mL of PRP was aspirated out and transferred into another tube for cell seeding.

### PRP gel/ADSCs complex

After cultured ADSCs reached confluent monolayer, they were collected by trypsin digestion and centrifuged at 1000 rpm for 5 min, ADSCs were washed 2 times with PBS to remove residual serum, then 0.3 mL cell suspension (2.0 × 10^6^) was resuspended in 0.1 mL PRP liquid from the same donor animal. To activate the PRP, 0.1 mL of calcium chloride (CaCl2) and bovine thrombin (Sigma, 1000 U/mL in 100 mg/mL CaCl2) mixture was added to ADSCs/PRP suspension to form a gel. Then put the PRP gel/ADSCs complexes into culture flask which contains 15 mL DMEM for coculture in vitro. The gels were divided into two groups: experimental group (PRP gel/ADSCs, *n* = 36) and control group (PRP gel, *n* = 36).

### Injectability evaluation

At the 8th week, the injectability of this scaffold/cell complex was investigated with the method in the study of Shamma et al. [18]. The performance of the complex during injection was compared with that of a market oily injection, Betolvex TM. A 5-mL syringe attached to a 19-gauge needle was filled with 1 mL of the tested material. An air pump was fixed to the syringe. For measurement of the injectability, air pump applied pressure to the complex surface. The pressure was measured in mmHg unit and maintained continuously at 100 mmHg using a sphygmomanometer. The time for release the 1 mL gel and the flow rate (mL/min) was recorded.

### Histological observation and cell viability detection

At the 2nd, 4th and 8th week, these complexes were examined by morphological and histological observation. The viability of cells in the PRP gel was measured with BrdU immunofluorescence method. Briefly, tissue slices were fixed with paraformaldehyde, permeabilized with 0.3% Triton X-100(Sigma), and blocked with 5% serum for 2 h at 4 °C. Before permeabilization, slices were pretreated with 2N HCl at room temperature for 30 min and washed 3 times. The slices were incubated with primary antibody (1/200) (Abcam) at 4 °C for 12 h. A goat polyclonal secondary antibody to rat IgG (FITC) (1/200) (Abcam) was used as secondary antibody. At last, the slices were analyzed by Fluorescence microscope (Olympus).

### Measurement of ultrastructure

To investigate the cell distribution of ADSCs/PRP complex, these complexes were prepared for scanning electron microscopy (SEM) test at the 2nd, 4th and 8th week. These complexes were washed three times in PBS(PH 7.4), fixed with 2% PBS-buffered glutaraldehyde at 4 °C for 2 h, post-fixed with 1% osmium tetroxide for 1 h, dehydrated in a graded ethanol series, replaced with gradient tert butyl alcohol, and sprayed with gold coating. Ultrathin sections (40–60 nm) were placed on grids (200 mesh). The grids containing the sections were observed on a TM-1000 SEM.

### Measurement of proteoglycan synthesis

The glycosaminoglycans(GAGs) in 3 ADSCs/ PRP gel complexes were measured with spectrophotometry as reported previously at 2nd,4th and 8th week [19]. Briefly, tissue wet weight for each complex was obtained, and each complex dried at 65 °C for 24 h. Dry weight was then obtained, followed by tissue digestion in 5 mg/mL proteinase K solution at 65 °C for 18 h. After digestion, each complex was analyzed for GAG content using the dimethylmethylene blue binding assay. GAG contents were normalized by tissue wet weight.

### Measurement of mRNA expression

Messenger ribonucleic acids (mRNA) of HIF-1α, aggrecan, type II collagen were determined by real-time polymerase chain reaction (RT-PCR). Total RNA was extracted from the retrieved cells with TRNzol (Invitrogen Biotechnology Co, Beijing, China) according to the manufacturer’s instructions. Then 1–2 μg of total RNA was reversely transcribed into cDNA in the reverse transcription (RT) system. For PCR, the system was designed as follows: 5 μl 10 × Taq buffer, 0.5 μl dNTP (10 mmol/L), 0.5 μl sense primer (20 μmol/L), 0.5 μl anti-sense primer (20 μmol/L), 2 μl cDNA, 0.5 μl Taq polymerase (Toyobo, Japan), adding ddH_2_O to 25 μl at 4 °C for 5 min; at 94 °C for 45 s; at 55 °C for 45 s; at 72 °C for 1 min; 30 cycles; at 72 °C for 10 min. Primers (Invitrogen Biotechnology Co, Ltd., Beijing, China, Table 1) based on rabbit sequences were designed and synthesized by integrated DNA technologies. After electrophoresis in 1.5% agarose gel with ethidium bromide alongside a 100 bp DNA ladder, the PCR product was visualized by a Bio-Imaging System (Bio-Rad).

**Table 1:** Primers sequence of target gene for RT-PCR

Genes	Primer sequence	Size (bp)
HIF-1α	Forward:5′- GTCGCTTCGGCCAGTGTG-3′	155
	Reverse:5′-GGAAAGGCAAGTCCAGAGGTG-3′	
Col II	Forward:5′-TCCCAGAACATCACCTACCA-3′	154
	Reverse:5′-CATCCTGCAGCACGGTATAG-3′	
Aggrecan	Forward:5′-GCTGCTACGGAGACAAGGAT-3′	107
	Reverse:5′-CTCACCCTCCATCTCCTCTG-3′	
GAPDH	Forward:5′-CCCTCAATGACCACTTTGTG-3′	117
	Reverse:5′-GGTTTGAGGGCTCTTACTCCT-3′	

### Statistical analysis

The data were described with $\overline{\mathrm{x}}$mean ± standard deviation. Statistical analysis was performed with SPSS 15.0 software package. Differences between groups were analyzed using one-way variance analysis (ANOVA) with post hoc LSD test. The *P* value less than 0.05 was considered statistically significant.

## Results

**Fig. 1:**
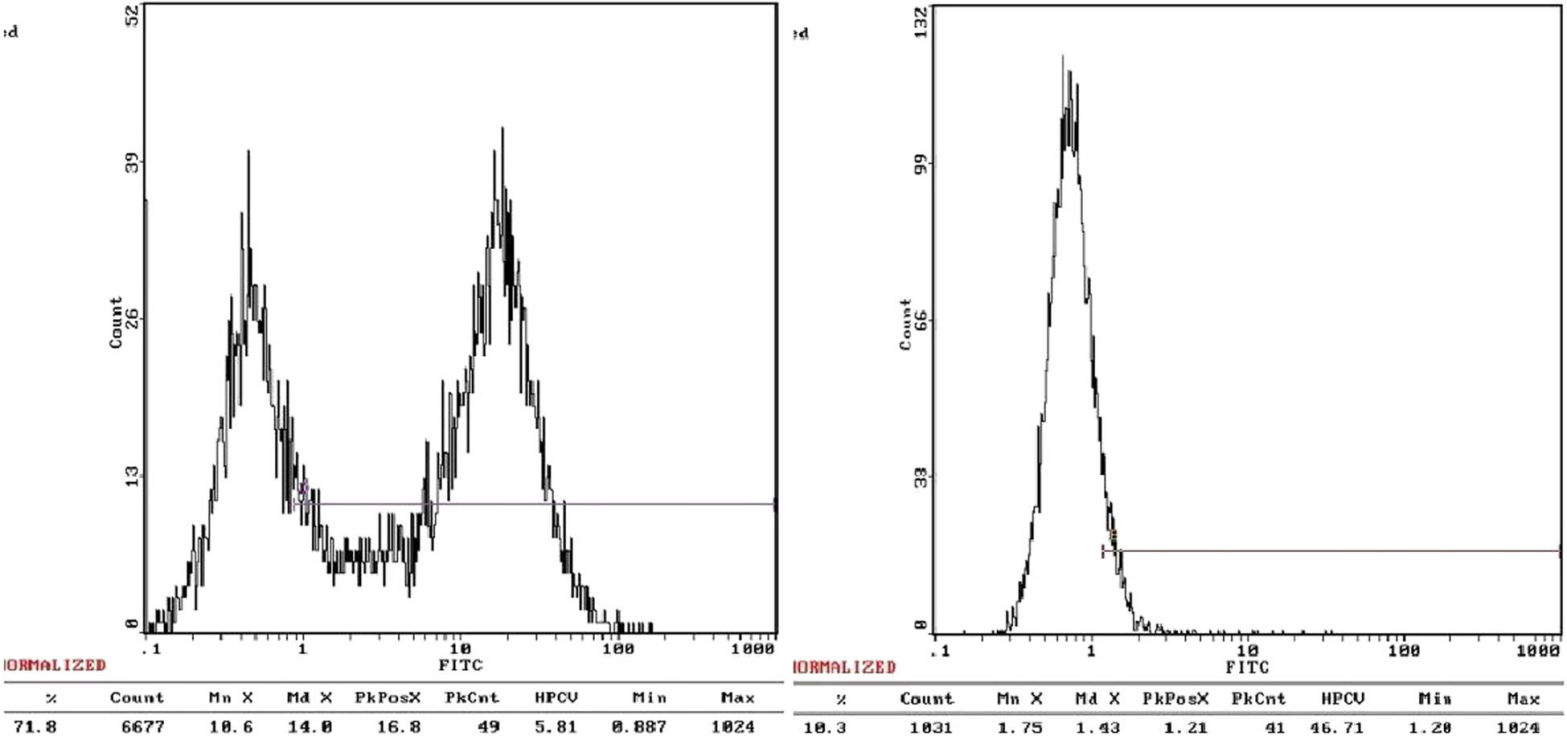
Flow cytometry analysis for third generation of ADSCs. CD90 were expressed at very high level (left), while CD45 at very low level (right)

Flow cytometry analysis showed that rabbit ADSCs expressed CD90 with 95.2% positive rate, which is a specific marker of stem cells. However, CD45 expression, which is lymphohematopoietic marker, was at very low level (Fig. 1). The results were in line with the phenotypes characteristics of ADSCs.

**Fig. 2:**
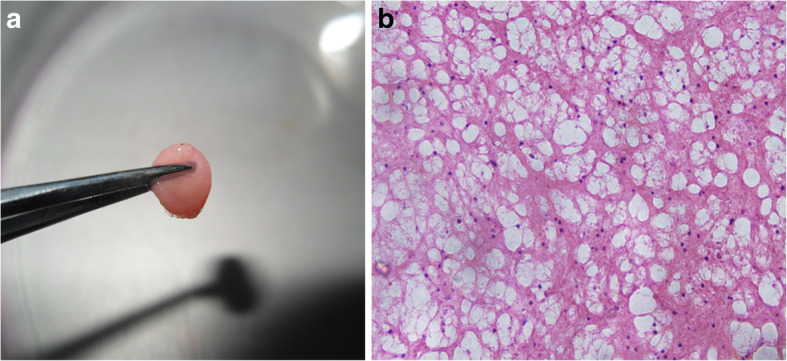
The gross view of PRP gel/ADSCs complex with semi-transparent pink, smooth and flexible surface, and good elasticity (**a**). HE staining (8 weeks): a large number of cells which had a blue-stained nucleus were well distributed in the reticulate PRP scaffold, × 400 (**b**)

**Fig. 3:**
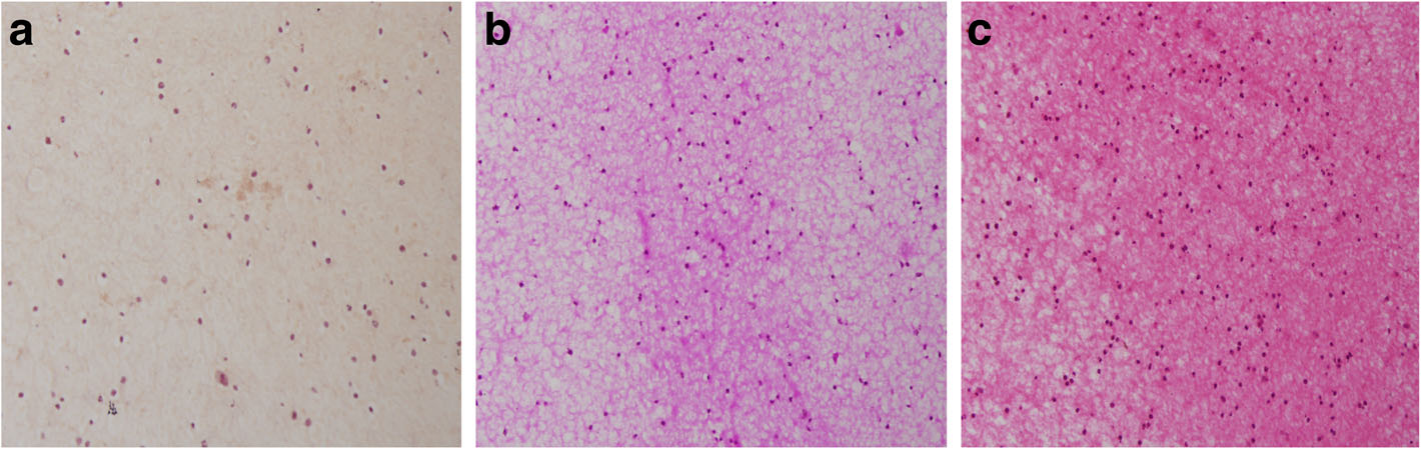
Safranin O staining. There was almost no positive pink dyeing extracellular matrix at 2nd week, × 200 (**a**). Pink dyeing was weakly positive in extracellular matrix at 4th week, × 200 (**b**). Positive pink dyeing was enhanced obviously in extracellular matrix at 8 weeks, × 200 (**c**)

**Fig. 4:**
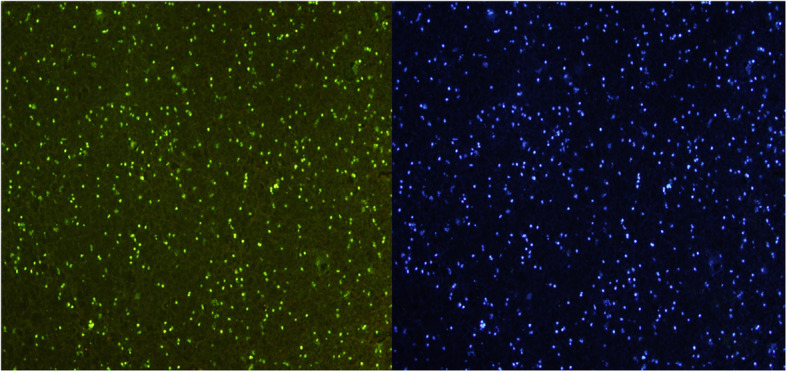
BrdU immumofluorescence staining. A large number of green fluorescent cell nucleus demonstrated the ADSCs can survive and proliferate in the PRP gel scaffold (8 weeks), × 400. The same view of DAPI restaining revealed the blue fluorescent cell nucleus, × 400

Macroscopically, the complex was solidified into gel with smooth surfaces and good elasticity at each time point (Fig. 2a). The results of HE staining demonstrated that the cells were well-distributed in the reticulate scaffold (Fig. 2b). Safranin O staining was used to investigate the existence of proteoglycans. The results confirmed almost no positive staining at 2nd week (Fig. 3a), very weak positive staining surrounding cells at 4th week (Fig. 3b). However, positive staining surrounding cells were enhanced obviously at 8th week (Fig. 3c). It was showed in BrdU immunofluorescence test that most ADSCs could survive and proliferate in the PRP gel scaffold at 8th week (Fig. 4).

**Fig. 5:**
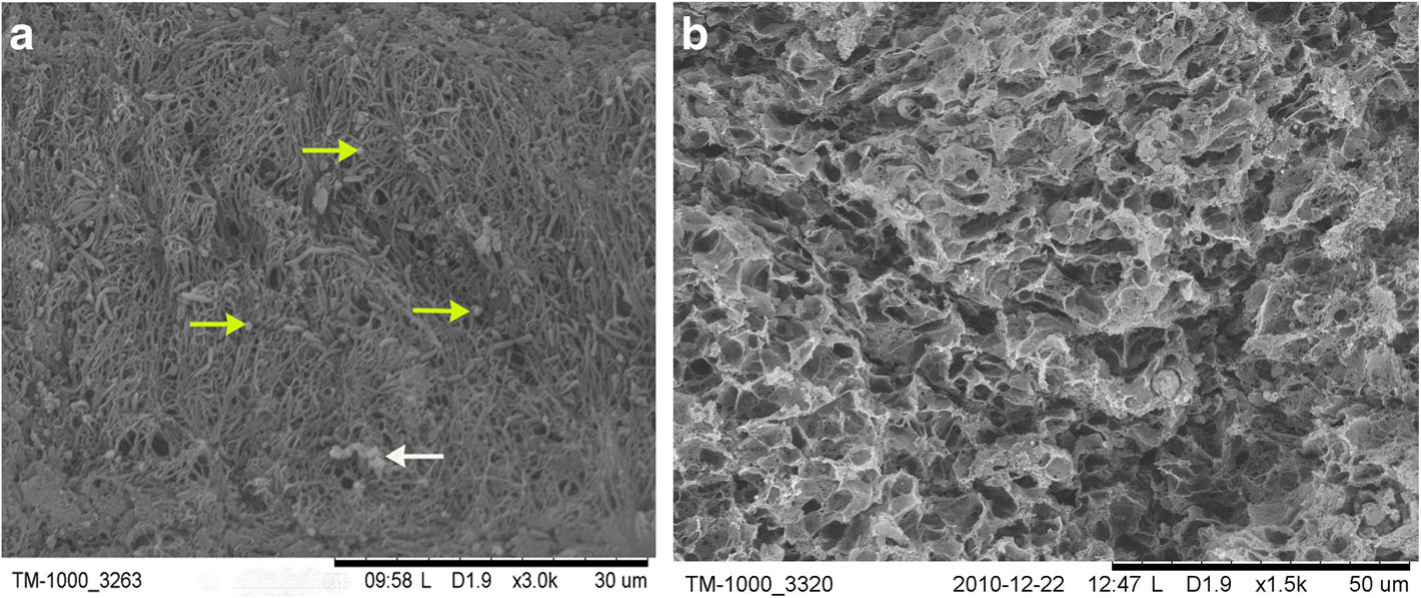
SEM showed the complexes had a network-like structure. It contained randomly arranged fibrillar elements of homogeneous thickness, with a large number of nucleus pulposus-like cells (yellow arrow) and a small amount of platelet aggregates (white arrow) distributed among them, × 3000 (**a**). It had a honeycomb-like and porous section. The pore size of PRP gel scaffold ranged from 5 μm to 100 μm, × 2000 (**b**)

The ultrastructure of PRP gel/ADSCs complex was demonstrated clearly with SEM images in which a three-dimensional network-like structure was revealed. It contained randomly distributed homogeneous reticulate fibrous structure in which there were nucleus pulposus-like cells differentiated from ADSCs and platelet adhesion (Fig. 5a). Meanwhile, it had a honeycomb-like and porous structure (Fig. 5b) with 5–100 um diameter of pores and 95% porosity. No binding elements were observed between the cells and filaments in fibrillar grid.

GAGs, main components of extracellular matrix in nucleus pulposus, were detected by spectrophotometry. The content of GAGs at 4th week was higher than those at 2nd week (*P* < 0.05). The significant differences were also noted between 4th and 8th week (*P* < 0.05).

**Fig. 6:**
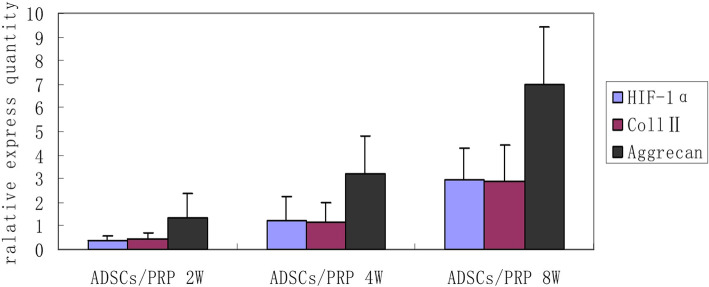
The results of RT-PCR test (relative express quantity) of ADSCs/PRP complex. The level of gene expression of HIF-1α, aggrecan, and type II collagen increase gradually with the increase of coculture time

The results of RT-PCR indicated that genes expression of HIF-1α, aggrecan, type II collagen at 4th week showed a significant increase than it at 2nd week (*P* < 0.05), and significant differences were also found between 4th and 8th week (*P* < 0.05) (Table 2, Fig. 6). Meanwhile, there were no positive staining and genes expression in the control group at each time point.

**Table 2:** The results of RT-PCR test (relative express quantity) of ADSCs/PRP complex

Genes	2 weeks	4 weeks	8 weeks
HIF-1α	0.38 ± 0.21	1.25 ± 0.98*	2.96 ± 1.35**
Coll II	0.47 ± 0.25	1.14 ± 0.86*	2.88 ± 1.52**
Aggrecan	1.35 ± 1.02	3.23 ± 1.58*	6.97 ± 2.47**

*Compared with 2nd (*P* < 0.05)

**Compared with 4th (*P* < 0.05)

The injectability of the PRP-gel/ADSCs complex was evaluated by the comparison of the mean flow rate with Betolvex^TM^ oil. The complex of PRP-gel/ADSCs took longer time to pass through the 19-gauge needle with the 100 mmHg pressure. The mean flow time for 1 mL Betolvex^TM^ injection was 3.011 ± 0.127 min with a flow rate of 0.332 mL/min. The flow rate 0.287 mL/min of complex was slower than that of oil.

## Discussion

PRP is a cocktail of concentrated autologous growth factors. Safety is one of the advantages of using autologous PRP for tissue regeneration because there is no need for concern about transmissible diseases or immunologic rejection [20]. Other advantages include the convenience of preparation and efficacy as a result of the synergistic effects from various endogenous growth factors. As an autologous injectable hydrogel, PRP gel provided a 3D environment for cultured ADSCs and can fix the seeded cells in injected site. Furthermore, application of injectable PRP gel has the characteristics of minimal invasion, better nutrition support and minimally immunological reaction in vivo. In addition, it has indicated that TGF-β promoted ADSCs differentiation towards a nucleus pulposus-like phenotype [15]. TGF-β and IGF-1 have strong effects on extracellular matrix production and proliferation of intervertebral disc cells [21]. Akeda and Chen et al. had reported that PRP may promote extracellular matrix synthesis metabolism of IVD cells in vitro [13, 22]. Therefore, we aimed to explore the feasibility of PRP gel as scaffold and ADSCs as seed cells, and to construct a new injectable tissue engineering nucleus pulposus in vitro.

In this study, the results of RT-PCR showed that genes expression of HIF-1a, aggrecan, type II collagen had significant increase along with culture time extending. Previous studies revealed that genes, such as collagen II, Sox-9, and aggrecan, were chondrocyte-specific [23]. Because the components of extracellular matrix in NP tissue are similar to that in articular cartilage, expression of several chondrogenic markers including SOX-9, aggrecan, and type II collagens have also been used as identification of NP cells [24]. Therefore, it was concluded that rabbit ADSCs had at least differentiated into chondrocyte-like cells in the PRP gel in this study. A successful tissue engineering NP graft should have similar structure and function to the native tissue. With current insufficient knowledge about NP biology, characterization of NP cells is still under being defined. Some cell markers such as hypoxia inducing factor-1 (HIF-1), glucose transporter-1 (GLUT-1), matrix metalloproteinase-2 (MMP-2), glypican 3 (GPC3), and keratin 19 (K19) are regarded as useful references [25, 26]. Particularly, in contrast to HIF-1β, HIF-1α was expressed only in the nucleus pulposus, and this is a form which was absent in both the cartilage end plate and annulus [27]. Therefore, gene HIF-1α can be considered to be a phenotypic character of the nucleus pulposus cells. It meant that ADSCs had differentiated towards a NP-like phenotype in our research.

Furthermore, we also performed Safranin O staining and spectrophotometry to examine the variation of GAGs in the extracellular matrix. Our study revealed the production of GAGs in PRP gel/ADSCs was higher at 8th week than that at 2nd and 4th week. It demonstrated that the change of protein level was consistent with the gene level. The results of HE staining and SEM demonstrated that the ADSCs were well-distributed and survived in the PRP 3-D reticulate scaffold in a long time. This result further confirmed that PRP gel could limit seeded cells in the injected site.

Although relatively new in stem cell research field, ADSCs has attracted intensive attention as a cell source in bone and cartilage repair. Particularly, application of ADSCs in NP tissue regeneration is greatly inspired in recent studies [28, 29]. Harvest of adipose tissue only involves a minimally invasive procedure that can be easily accepted and performed in outpatient clinics, and the number of adherent cultured ADSCs can reach up to 25,000/g of adipose tissue. Therefore, according to easy procedure and considerable amount of cells, adipose tissue could serve as a suitable source of stem cells for clinical applications.

Undoubtedly, the injectability of scaffold is an important advantage. Injectable scaffold/cell complexes via minimally invasive approach are attractive because they could be injected through a needle under fluoroscopy into the degenerative disc space easily. In our previous study [30], the discogenic low back pain was dealt with by percutaneous nucleoplasty using coblation technique with 19-gauge needle. Accordingly, the key parameters for injectability, the flow time, and flow rate were tested with 19-g needle. Although the flow rate of our complex was not better than market product, the injectability was confirmed in this experiment at least. The collagen concentration might be attributed to the increase in viscosity and the low flow rate.

Based on the philosophy of autologous PRP gel/ADSCs, venous blood is the best choice clearly. Arterial blood sampling is more difficult and risky, requiring more time to control bleeding by compression, causing more discomfort to patients. Therefore, venous blood was selected in almost all clinical studies. However, in this research, the peripheral blood was drawn from rabbit’s central ear artery, just because the blood collection from central ear artery is easier than marginal ear vein, and the blood collection volume is larger. That is why we use arterial blood in this research. In fact, there have been very few studies comparing platelet activity in venous and arterial blood, and there have been no studies comparing PRP preparation with venous and arterial blood. This research [31] showed that platelet activity was elevated when platelets were derived from arterial blood. Although the activity of platelets in arterial blood may be higher than that in venous blood, there are great limitations in clinical application.

Biomechanical factors also play a very important role on cell in nucleus pulposus. The morphological structure and function of NP cells are all related to the mechanical environment. Periodic tensile stress may promote the proliferation of nucleus pulposus cells and collagen synthesis. Moreover, the mechanical environment may promote the stem cells differentiation to NP-like cells or make the NP-like cell synthesis more extracellular matrix [32]. This research did not do the experiments related to mechanics. This is really a shortcoming of our research. Further researches of in vivo and in some mechanical environment in vitro, such as static pressure, should be carried out.

In previous researches, it was seldom reported to explore the feasibility of PRP gel as scaffold and ADSCs as seed cells to construct injectable tissue engineered nucleus pulposus. Although successful reconstruction of tissue engineered nucleus pulposus in vitro in this experiment, the effectiveness of application in vivo with this kind of injectable tissue engineered nucleus pulposus to repair the intervertebral disc degeneration should be tested in an experimental animal model in further studies.

## Data Availability

The datasets collected and/or analyzed in the current research available from the corresponding author on reasonable request.

## Abbreviations

PRP: Platelet-rich plasma
ADSCs: Adipose-derived stromal cells
BrdU: Bromodeoxyuridine
GAG: Glycosaminoglycans
HIF-1α: Hypoxia inducible factor-1 alpha
RT-PCR: Reverse transcription-polymerase chain reaction
SEM: Scanning electron microscope
MSCs: Mesenchymal stem cells
PDGF: Platelet-derived growth factor
TGF-β1: Transforming growth factor β1
IGF: Insulin-like growth factor
PBS: Phosphate-buffered saline
DMEM: Dulbecco’s modified Eagle’s medium
FBS: Fetal bovine serum
FACS: Fluorescence activated cell sorting
FITC: Fluorescein isothiocyanate
EDTA: Ethylenediaminetetraacetic acid
PPP: Platelet-poor plasma
ANOVA: Analysis of variance
IVD: Intervertebral disc
NP: Nucleus pulposus
MMP-2: Matrix metalloproteinase-2
GLUT-1: Glucose transporter-1
GPC3: Glypican 3
K19: Keratin 19
